# *FUNCTION* Is One Year Old: How Did We Do?

**DOI:** 10.1093/function/zqab023

**Published:** 2021-04-23

**Authors:** Ole H Petersen

**Affiliations:** School of Biosciences, Cardiff University, Cardiff, CF10 3AX, Wales, UK

It is now almost exactly one year since my inaugural editorial was published,^1^ so it may be a good time to review what *FUNCTION* has achieved so far. We had very ambitious plans.^1^ Did we succeed in starting out on the path we had signposted?

The primary aim we announced was to publish the best of physiology and pathophysiology and to place new and important findings in a general context by publishing a Perspective (commentary) related to each original research article. We also wished to give as much exposure as possible to important new original research findings published in *FUNCTION* by giving opportunities for authors to present their findings at meetings of the American Physiological Society (APS). We announced that we would do our best to speed up the publication process and also, at the same time, contribute to improved reliability by avoiding as much as possible the so-called “reviewer experiments,” making a firm decision about acceptance or rejection at the end of the first review round, even when some revision was needed.^1^

It is clearly impossible, at this early stage in the life of the journal, to judge to what an extent we managed to attract outstanding articles, as time is probably the most important “filter” in this respect, but we have certainly published original research articles from many of the most prominent investigators in the fields of physiology and pathophysiology. Naturally, we hope that the articles we have published and will be publishing, will become highly cited, but it is not the primary aim of *FUNCTION* to obtain a high Impact Factor. If this were to be our overriding goal, we should currently focus on publishing review articles dealing with COVID-19! In fact, we have been happy, and shall continue to be happy, to publish on any topic within the broad fields of physiology and pathophysiology and it has been gratifying to see that many of these articles have actually already been well cited, considering the short period in which we have been functional. In deciding what to accept or reject, we shall continue to be guided by answers to the three classical questions: “Is it new?,” “Is it important?,” and “Is it true?,” but also by “Is it well written?.”

As one of the authors of the 2020 ARRIVE guidelines for the reporting of animal research,^2^ I naturally believe, as stated in our article, that “Transparent reporting of research methods and findings is an essential component of reproducibility.”^2^ However, the majority of cases of irreproducible results in my own research field have actually been caused by use of techniques that were unsuited to the experimental tasks carried out and/or were insufficiently mastered.^3^ Whereas it is relatively easy to use a checklist to satisfy oneself that all required elements have been adequately reported in a submitted manuscript, it is of course more difficult to judge the degree of experimental skill by the authors and the suitability of the methodological approach. Experienced editors, able to secure the services of the most appropriate specialist reviewers, are needed.

A distinguished colleague of mine once told me that whenever he submits a paper or a grant application, he “prays” for the assessment to be undertaken by mature individuals. I believe this is the case for *FUNCTION*. The journal has assembled an outstanding group of very experienced editors, who have all made their mark in science and therefore have nothing to prove. The inaugural key group of Executive Editors has very recently been expanded by the appointment of Donald Bers (University of California, Davis) who has made so many important and impactful contributions to our knowledge of cardiovascular physiology and pathophysiology. I had the pleasure and honor to work with Don when we were both Senior Editors of the *Journal of Physiology* and now look forward to working with him again.

It is my personal view that ultimately authors are responsible for the content of their papers and although *FUNCTION* has to be satisfied that the data presented are novel, important, and likely to be reliable, I do not think that we (editors/reviewers) should dictate the opinions authors are allowed to express. Unlike many other journals, we have not gone back to authors several times with further revision requirements. Generally, papers in *FUNCTION* only go through a single revision round.

We have fulfilled our promise to publish quickly. In 2020, the mean time from submission to the decision point (at the end of the first review round) was 16.3 days and time from acceptance to final publication was 1.1 months. Accepted manuscripts have been posted on our website (Advance Articles) within 72 h after the principal author signed the license agreement.

We have managed to publish perspectives in relation to almost every single original research article, although in several cases it turned out not to be feasible to publish the perspective in the same issue as the original article, as this would have imposed a delay on the final publication of the original research.

Our new style Evidence Reviews have done well, both with regard to downloads and citations. The restrictions imposed by *FUNCTION* on authors of these reviews may be regarded as relatively severe (all factual statements can only be supported by references to original papers and not to other review articles) and some authors have found this challenging, but also innovative and invigorating. We would like to publish more of these articles, as we think they provide a valuable new perspective on clarifying precisely what has actually been shown in a particular field.

One disappointment so far, has been the relatively few submissions of papers in the Function Focus category. These papers are relatively short original research reports of significant and fully documented findings for which we do not require exploration of all the ramifications that typically are expected for full papers. So far, we have only published three such papers^4–6^ (and, at the time of writing this editorial, the last one has only just appeared in Advance Articles^6^). Our two first Function Focus articles^4^^,^^5^ have done remarkably well with regard to both downloads and citations. We therefore hope that others will now see that this category offers a valuable and very visible opportunity to signpost new and important findings after fast peer review.

The principal aims of journals and meetings are the same, namely to facilitate communication between scientists, and we have used the opportunities for *FUNCTION* events (webinars and participation in the APS Annual Meeting) to give additional publicity to papers published in *FUNCTION*. So far, *FUNCTION* has organized four webinars plus a symposium at EB2021. *FUNCTION* authors are given preference with regard to invitations to talk at these events and, so far, ∼30% of the papers published in the journal have been presented on these occasions. When we eventually get out of the COVID-19 crisis, we do of course hope and expect to organize many physical *FUNCTION* events, but shall no doubt also continue to utilize the very efficient webinar format which, in our case, has repeatedly attracted audiences of ∼400.

At the end of this first year of publishing, I wish to thank my editorial colleagues, who have generously given priority to evaluating manuscripts for *FUNCTION* and, importantly, also very visibly demonstrated their support for this new journal by contributing some of their best papers. I am also very grateful to the many authors from outside the editorial groups, who have shown confidence in the journal by submitting excellent papers to us. This bodes well for the future of *FUNCTION*.

## Funding

The author acknowledges support from the European Commission’s Horizon 2020, grant agreement 737432.

## Conflict of Interest Statement

O.H.P. is Editor-in-Chief of *Function*.
